# The Effects of Packaging Barrier Properties Coupled with Storage Temperatures on the Dominant Spoilage Bacteria Composition and Freshness Quality of Lamb

**DOI:** 10.3390/foods14030343

**Published:** 2025-01-21

**Authors:** Debao Wang, Xiaoyu Chai, Su Wang, Tongtong Zhao, Xiaochun Zheng, Weili Rao, Huiguo Yang, Dequan Zhang, Chengli Hou

**Affiliations:** 1 Institute of Food Science and Technology, Chinese Academy of Agricultural Sciences, Key Laboratory of Agro-Products Quality & Safety in Harvest, Storage, Transportation, Management and Control, Ministry of Agriculture and Rural Affairs, Beijing 100193, China; wangdebao@caas.cn (D.W.); 15027708107@163.com (X.C.); wangsu0630@163.com (S.W.); zhengxiaochun@caas.cn (X.Z.); dequan_zhang0118@126.com (D.Z.); 2 Institute of Agricultural Product Processing and Nutritional Health, Chinese Academy of Agricultural Sciences (Cangzhou), Cangzhou 061019, China; ztt940226@163.com; 3 College of Food Science and Technology, Hebei Agricultural University, Baoding 071000, China; iamraoweili@126.com; 4 Xinjiang Academy of Animal Science, Urumqi 831399, China; yanghuiguo3239@163.com

**Keywords:** lamb, high-oxygen-barrier packaging, dominant spoilage bacteria, storage temperature, shelf life

## Abstract

This study aims to establish a preservation method by coupling certain barrier packaging with storage temperatures suitable for extending the shelf of chilled lamb. Chilled lamb was packaged using three different oxygen permeability packaging materials of high-oxygen-barrier packaging (HORP), medium-oxygen-barrier packaging (MORP), and low-oxygen-barrier packaging (LORP) (1.70, 23.95, and 1631.44 cm^3^/(m^2^·24·h·0.1·MPa), respectively, then stored at temperatures of 4 °C and −1 °C for 28 days, respectively. The results of total viable count, pH, color, and volatile basic nitrogen indicate that HORP effectively inhibits the growth rate of surface microorganisms and the oxidation rate of proteins in lamb. The sulfhydryl content, carbonyl value, and electronic nose suggest that the oxidative decomposition rate of lamb during storage at −1 °C is lower compared to storage conditions at 4 °C. The microbial diversity suggests that HORP significantly hinders the growth and reproduction of *Pseudomonas* and *Brochothrix* aerobic spoilage bacteria, as well as diminishes the abundance of the dominant microbial community. Herein, utilizing high-barrier packaging with an oxygen permeability of lower than 1.70 cm^3^/(m^2^·24·h·0.1·MPa) in conjunction with ice temperature storage at −1 °C is a highly effective preservation method for prolonging the shelf life of chilled lamb to 28 days.

## 1. Introduction

The consumption of lamb, a protein-rich meat with low fat content and abundant vitamins, provides essential amino acids, fatty acids, and other nutrients necessary for the human body [1]. With the evolving consumption patterns and growing awareness towards healthy eating, ensuring meat quality and safety and preserving freshness have emerged as key focus areas [2]. Packaging serves as an effective barrier, effectively safeguarding meat and other food products against physical, chemical, and biological factors, which ensures the preservation of food quality and extends shelf life [3,4,5]. However, the selection of packaging for chilled lamb preservation faces two challenges: firstly, the permeability of the packaging material used is unclear; secondly, the optimal storage temperature for preserving chilled lamb remains uncertain. Therefore, a preservation method of certain barrier packaging coupled with a storage temperature suitable for extending the shelf of chilled lamb needs to be addressed.

The barrier properties of packaging typically refer to the packaging material’s capacity to effectively impede the permeability of specific substances (such as gases and liquids) from one side to the other [6]. For meat packaging, it is crucial to explore the water and oxygen barrier performance that matches the required grade for preserving the quality and extending the shelf life of meat, taking into account differences in the quality of livestock and poultry products [6,7]. Oxygen is the key factor that causes meat products to oxidize and regulates the decline of spoilage microorganisms, so low-oxygen-permeability packaging will be the inevitable choice for meat preservation packaging [8]. A study reported that using vacuum packaging with different oxygen permeabilities (<10, 3000, ≥7000 cm^3^·m^2^·24 h) for chicken legs effectively reduced the microorganism count in the product [8,9]. Wu et al. (2023) investigated the effects of dual-layer intelligent packaging films based on guar gum/modified anthocyanin and carboxymethyl cellulose sodium/starch/Nisin on the preservation of chicken breast meat, and the results showed that enhancing the barrier properties of the packaging materials can effectively ensure the quality of chicken breast meat and extend the shelf life [10]. Hernández-García et al. (2022) developed a biodegradable, eco-friendly, and sustainable polylactic acid packaging film with oxygen and water vapor permeability values of 11.54 × 10^14^ m^3^·m^−2^·Pa^−1^·s^−1^ and 92.59 × 10^11^ kg·m^−2^·Pa^−1^·s^−1^, respectively [11]. However, despite these properties, when compared to high-barrier commercial packaging films with water and oxygen permeabilities both below 2.5 kg·m^−2^·Pa^−1^·s^−1^ (m^3^·m^−2^·Pa^−1^·s^−1^), the chilled pork slices packaged using this film exhibited elevated levels of lipid and protein oxidation and a noticeable darkening in color, as well as dehydration [11].

Low-temperature storage is a crucial factor in ensuring the quality of meat products, effectively preserving meat quality by inhibiting microbial growth, reducing enzyme activity and water loss, slowing down oxidation reactions, and preventing contamination through various mechanisms [12]. The active film developed by Guo et al. (2023) consists of pectin and polyphenols, which, when combined with ultra-cryogenic storage, effectively inhibit protein and fat oxidation while preventing color deterioration in lamb [13]. The study by Coombs et al. (2017) has demonstrated that chilling and freezing both have positive and negative effects on the quality attributes of red meat, including tenderness, juiciness, flavor, color, and microbial growth [14]. Chilling enhances meat tenderness, flavor, and color; however, this improvement is dependent on maintaining taste and color below the threshold for deterioration while preventing microbial spoilage from reaching harmful levels [14]. Freezing results in significant juice loss during thawing, leading to economic losses as well as a decrease in tenderness and sensory quality [14]. In summary, in order to cater to the public’s consumption habits and promote the development of the industry, it is imperative to develop a flexible combination of packaging with a certain level of barrier properties and specific storage temperature for preserving lamb.

The present study investigated the impact of various barrier packaging materials in combination with storage temperatures of 4 °C and −1 °C on the quality attributes of chilled lamb. By assessing the total viable count, pH level, protein oxidation, sensory attributes (including color and odor), water holding capacity, and microbial diversity of lamb during storage, the objective was to elucidate a specific grade of oxygen-barrier, high-barrier packaging combined with low-temperature storage for effectively preserving and minimizing losses in chilled lamb.

## 2. Materials and Methods

### 2.1. Materials

Longissimus dorsi muscles of six sheep (Gangba Tibetan sheep, 36 months old) were purchased from Shigatze Qomolangma Nongtou Baiyacheng Agricultural and Animal Husbandry Products Processing Co., Ltd. (Lasa, China). The packaging materials were provided by Sunrise Material Co., Ltd. (Jiangyin, China). Plate counting agar (pH 7.0 ± 0.2) was purchased from Landbridge Technology Co., Ltd. (Beijing, China). HCl (0.01 mol/L) and carbamid were obtained from Sinopharm Chemical Reagent Co., Ltd. (Shanghai, China). Trichloroacetic acid was bought from Aladdin Biochemical Technology Co., Ltd. (Shanghai, China). 2,4-dinitrophenylhydrazine hydrochloric acid buffer was purchased from Qiyan Biotechnology Co., Ltd. (Beijing, China).

### 2.2. Experimental Design

The experiment comprised of six experimental groups, wherein the packaging materials utilized were high-oxygen-barrier packaging bags (HORP, nylon/ethylene-vinyl alcohol/Polyethylene), medium-oxygen-barrier packaging (MORP, polypropylene/PE), and low-oxygen-barrier packaging (LORP, polyethylene) with oxygen barrier rates of 1.70, 23.95, and 1631.44 cm^3^/(m^2^·24 h·0.1 MPa), respectively, and the selected storage temperatures were either 4 °C or −1 °C, resulting in the following groupings: CHORP, CMORP, and CLORP for lamb packed with HORP, MORP, and LORP packaging materials stored at 4 °C; FHORP, FMORP, and FLORP for lamb packed with HORP, MORP, or LOPR packaging materials stored at −1 °C. The samples consisted of longissimus dorsi muscles from six Gangba Tibetan sheep, namely *n* = 6. All stored samples were collected at 0, 7, 14, 21, and 28 d. Following sampling, immediate determinations were made for total viable count (TVC), total volatile basic nitrogen (TVB-N), pH value, color, water phase state, and electronic nose analysis. The remaining samples were frozen and stored at −80 °C for subsequent determination of oxidation indicators and the trend of predominant spoilage bacteria.

### 2.3. Total Viable Count (TVC)

The meat sample weighing 5.00 g was accurately measured and placed in 45 mL of sterile saline solution following the determination method according to GB 4789.2-2022 [15]. The sample was subjected to uniform agitation for 5 min, followed by absorption of 1 mL and the subsequent addition to 9.00 mL of sterile saline for a tenfold dilution. After dilution, three suitable gradients were chosen, each consisting of three parallel gradients. A total volume of 100 μL from each gradient was absorbed and coated onto a plate, followed by incubation at 37 °C for 24 h.

### 2.4. pH Value and Color

The pH value of each meat sample was measured by using a hand-held portable pH meter (Testo 205, Testo, Lenzkirch, Germany). The color of the meat sample was determined at 25 °C following the opening of the packaging using a D65 colorimeter (CM-600d, Konica Minolta, Tokyo, Japan). Prior to measurement, it was necessary to calibrate the color difference meter. The L*, a*, and b* values of the meat sample were recorded.

### 2.5. Water Phase Change

The water phase composition of meat samples was determined using a hydrogen proton nuclear magnetic resonance imaging system (NMI20-040H-I, NIUMAG, Suzhou, China). The meat sample was cut into cubes measuring approximately 1 cm × 1 cm × 2 cm with flat and vertical sections. Transverse relaxation time (T_2_) was measured with a CPMG sequence. Experimental conditions included a proton resonance frequency of 20 MHz, a 10.00 μs pulse duration for the 90° pulse, a 19.52 μs pulse duration for the 180° pulse, six repeated samplings (*n* = 6), a repetition interval of 1500.00 ms (TW = 1500.00 ms), a total of 3000 echoes acquired (NECH = 3000), and a sampling frequency of SW = 100 kHz.

### 2.6. Total Volatile Base Nitrogen (TVB-N)

The TVB-N content of the meat sample was determined in accordance with GB 5009.228-2016 [16]. The meat sample, weighing 5.00 g, was accurately measured and placed into a 25 mL solution of trichloroacetic acid with a concentration of 20 g/L, followed by homogenization for 20 s. The homogenized sample solution was incubated in an ice bath for 30 min and subsequently filtered. The filtrate (10 mL) and magnesium oxide (1.0 g) were simultaneously introduced into the protein digestion tube, which was promptly connected to the Kjeldahl nitrogen analyzer for determination. In the distillation extraction process, 10 mL of boric acid solution (20 g/L) was added, followed by a distillation time of 5 min and a subsequent leaching water volume of 10 mL. In the final titration, a mixture of distilled liquid and 5 drops of indicator was titrated using 0.01 M HCl, and the titration volume was recorded.

### 2.7. Total Sulfhydryl Content

The method for evaluating protein oxidation in chilled lamb was adopted based on the approach proposed by Gao et al. [17]. The 1.00 g sample was weighed and subsequently homogenized with 10 mL of a 2% (*w*/*v*) SDS buffer at a speed of 10,000 rpm three times, each lasting for 30 s. Following this, the mixture was centrifuged at 4 °C with a force of 4000× *g* for 20 min to obtain the supernatant. The protein concentration was adjusted to 2.00 mg/mL using the BCA protein kit. The protein solution (0.50 mL) was combined with Tris-Gly-8 Murea buffer (2.50 mL) and 5,5′-disulfide di-2-nitrobenzoic acid (DTNB) solution (0.02 mL of 4.00 mg/mL). The mixture was then incubated at 25 °C for 30 min, followed by measurement of the absorbance at 412 nm (A412). The total sulfhydryl content (-SH μmol/g protein) was calculated using the following formula:

(1) $$-\mathrm{SH}\;(\mu \mu \mathrm{mol}/\mathrm{Pro})=\frac{75.53\times A_{412}\times D}{C}$$

where D is the dilution coefficient (6.04), and C is the concentration of protein in the test sample.

### 2.8. Carbonyl Content

A 200.00 μL solution of total protein at a concentration of 2.00 mg/mL was added to a 10% (*w*/*v*) trichloroacetic acid (TCA) solution, followed by centrifugation at 12,000 rpm for 5 min at 4 °C. The resulting supernatant was discarded, followed by vortexing the mixture with 2,4-dinitrophenylhydrazine to initiate a 30 min dark reaction. After another round of centrifugation, the supernatant was decanted, and a TCA solution was added. Subsequently, an ethanol–ethyl acetate solution was introduced to facilitate precipitation. The insoluble substances in the precipitate were removed using a phosphate buffer, and absorbance measurements were taken at wavelengths of both 280 nm and 370 nm after a duration of 15 min.

(2) $$\mathrm{Carbonyl}\;\mathrm{content}\;(\mathrm{nmol}/\mathrm{mg}\;\mathrm{Pro})=\frac{A_{370}}{22,000\times (A_{280}-A_{370}\times 0.43)}\times 10^{6}$$

### 2.9. Volatile Odor

The ground meat sample should be precisely weighed at 2.00 g and carefully transferred into a designated flavor bottle. Subsequently, the response degree of the sample to the sensor array needs to be meticulously analyzed, with data collected specifically from the time interval between 48 and 52 s being selected for further comprehensive analysis.

### 2.10. Microbial Community Characterization

The bacterial solution obtained from different meat samples during storage was centrifuged at 4 °C for 10 min (2000 revolutions per min). The initial centrifugation was conducted, followed by the absorption of the supernatant at 4 °C and 12,000 r/min for a second centrifugation. Subsequently, the sediment was collected and stored in a refrigerator at −80 °C. The V3–V4 region of bacteria was amplified using the Illumina Miseq high-throughput sequencing platform, with primer 338F (5′-ACTCctacgggaggcagcagg-3′) and 806R (5′-GGACTACNNGGGTATCTAAT-3′). The samples were sequenced, and operational taxonomic units (OTUs) were assigned based on a similarity threshold of 97% to determine the species present. The OTU and Silva data bank were utilized for measuring purposes, in order to obtain corresponding species diversity information. Alpha diversity indices (Chao1, Observed_species, PD_whole_tree, and Shannon) were calculated and analyzed by combining the counts. Additionally, the UniFrac algorithm was employed to analyze the differences in microbiota composition and Beta diversity.

### 2.11. Statistical Analysis

The statistical analysis of all data was conducted using SPSS 27.0 software. ANOVA and the least-square method were employed to examine the impact of packaging barrier coupled with storage temperature on all variables at a significance level of *p* < 0.05. Data were shown as mean ± standard deviation (SD). The figures were created using Origin 18C. The graphical representations of the microbial flora were generated using the Microbiome Analyst system provided by Shanghai Meiji Biomedical Technology Co., Ltd. (Shanghai, China).

## 3. Results and Discussion

### 3.1. Effects of Packaging Barrier Coupled with Storage Temperature on TVC in Chilled Lamb During Storage

**Figure 1:**
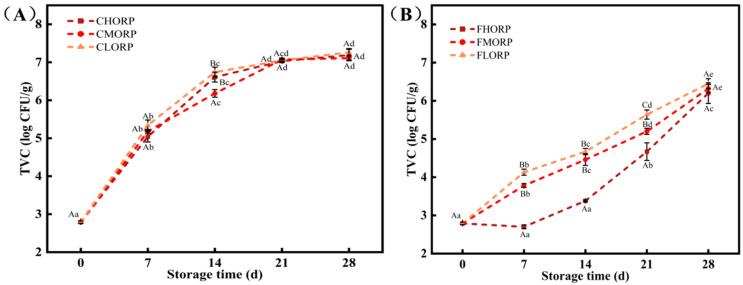
Changes in TVC of chilled lamb under different barrier packaging during storage at 4 °C (**A**) and −1 °C (**B**). Different small letters (a, b, c, d, e) indicate a significant difference (*p* < 0.05) in storage time; at the same time points, different capital letters (A, B, C) indicate a significant difference (*p* < 0.05) among the treatments.

The main culprits behind meat spoilage are the insidious contamination by microorganisms and their rapid growth [18,19]. As shown in Figure 1A, the growth rate of microorganisms on the surface of chilled lamb packaged using three types of oxygen permeability materials is as follows: LORP < MORP < HORP. The TVC growth rate of the chilled lamb stored at −1 °C was significantly lower than that stored at 4 °C for all three groups. The preservation method of HORP packaging combined with −1 °C storage temperature significantly inhibits TVC in chilled lamb. The findings are consistent with the results reported by Kartika et al. [9] and Hernández-García et al. [11]. During 0–7 d of storage at 4 °C, the TVC of the CLORP, CMORP, and CHORP groups increased from 2.79 log CFU/g to about 5.00 log (CFU/g). After 14 d of storage at 4 °C, the TVC in the CHORP, CMORP, and CLORP groups exceeded the maximum limit for microbial count in chilled meat set at 6.00 log CFU/g as specified in NY/T 632-2002 [20] with measurements of 6.61, 6.18, and 6.74 log CFU/g, respectively. However, after being stored at −1 °C for 21 days, the TVC in the FHORP, FMORP, and FLORP groups was measured as 4.67, 5.20, and 5.64 log CFU/g, respectively, as shown in Figure 1B. After being stored at −1 °C for 21 days, the TVC in the HORP was only 6.20 log CFU/g. Therefore, the preservation method of high-barrier packaging combined with storage at −1 °C can effectively inhibit the growth and reproduction of microorganisms in chilled lamb.

### 3.2. Effects of Packaging Barrier Coupled with Storage Temperature on Microflora Structure in Chilled Lamb During Storage

**Figure 2:**
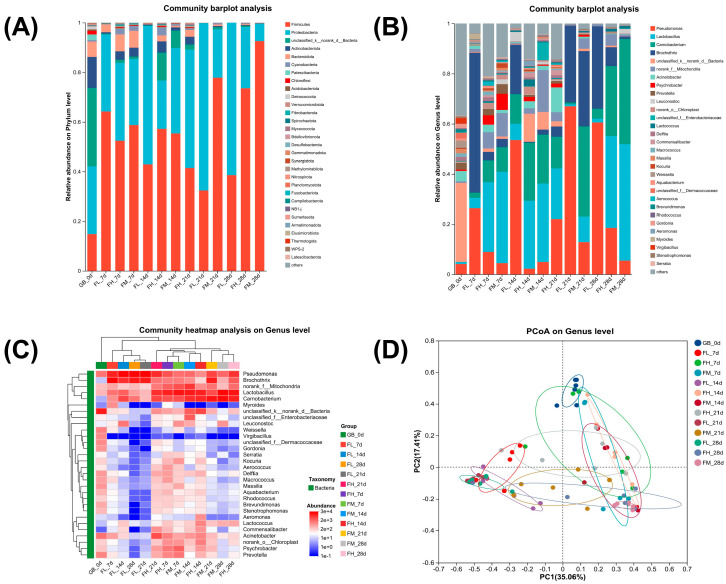
Changes rule of bacterial flora of chilled lamb under different barrier packaging. (**A**) Community abundance on the phylum level; (**B**) Community abundance on the genus level; (**C**) Community heatmap on the genus level; (**D**) PCoA on the genus level.

Accurately determining the predominant spoilage bacteria composition in chilled meat and understanding their growth and decline patterns during storage are crucial for selecting appropriate meat packaging and conducting research on preservation and loss-reduction technologies. The predominant bacterial composition responsible for spoilage in chilled lamb meat under different barrier packaging conditions during storage is illustrated in Figure 2. The main spoilage bacteria in meat during storage, as depicted in Figure 2A, are firmicutes and proteobacteria. The results of further identification indicated that *Pseudomonas*, *Carnobacterium*, *Lactobacillus*, and *Brochothrix* were the predominant spoilage bacteria observed during the storage of chilled lamb, as depicted in Figure 2B,C. These findings are consistent with the research findings reported by Cauchie et al. [21] and Lauritsen et al. [22]. The abundance of bacterial flora exhibited significant variations among the HORP, MORP, and LORP groups. As depicted in Figure 2B–D, in contrast to LORP, which exhibited an upward trend of pseudomonas during storage, both HORP and MORP demonstrated a pronounced inhibitory effect on *Pseudomonas*, effectively maintaining a lower level of *Pseudomonas* throughout the entire storage period. In comparison to the high abundance of *Brochothrix* flora observed in the LORP group, both HORP and MORP groups consistently exhibited significant inhibition of *Brochothrix* throughout the entire process. The abundances of *Lactobacillus* and *Carnobacterium* were found to be significantly higher in the HORP and MORP groups. The aforementioned statement further demonstrates that high-barrier packaging effectively hinders the growth activity of aerobic bacteria [23,24]. However, based on observed changes in the freshness of chilled lamb under three different barrier-packaging conditions, the predominant bacterial species responsible for lamb spoilage were determined to be *Pseudomonas* and *Brochothrix*, which are obligate aerobic bacteria. Therefore, for the preservation of lamb, it is imperative to opt for high-oxygen-barrier packaging with an oxygen permeability below 1.70 cm^3^/(m^2^·24 h·0.1 MPa).

### 3.3. Effects of Packaging Barrier Coupled with Storage Temperature on pH Value in Chilled Lamb During Storage

**Figure 3:**
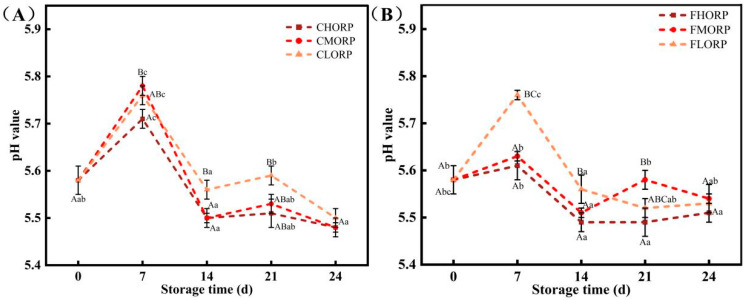
Changes in pH value of chilled lamb under different barrier packaging during storage at 4 °C (**A**) and −1 °C (**B**). Different small letters (a, b, c) indicate a significant difference (*p* < 0.05) in storage time; at the same time points, different capital letters (A, B, C) indicate a significant difference (*p* < 0.05) among the treatments.

The pH value is also a crucial indicator for assessing the freshness of meat, as the presence of alkaline substances like ammonia and amines generated during meat spoilage can significantly impact pH changes during storage [25]. The effects of packaging barriers coupled with storage temperature on the pH of chilled lamb are shown in Figure 3. The pH of chilled lamb in each treatment group tended to initially increase and subsequently fluctuate downwards with prolonged storage time. The initial pH of chilled lamb on day 0 was measured to be 5.58. The pH values of lamb in the CHORP, CLORP, and CMORP groups were the highest (*p* < 0.05) on the 7th d and were 5.71, 5.78, and 5.76, respectively. The pH value of chilled lamb exhibited a declining trend during the storage period of 7–28 d. The changes observed in the three groups at −1 °C were consistent with those observed at 4 °C. The pH values of the HORP and MORP groups, as illustrated in Figure 3, demonstrated a comparatively slower rate of both increase and decrease when compared to that observed in the LORP group. The high- and medium-oxygen-barrier packaging materials effectively hindered the metabolic activity of microorganisms and protein degradation in lamb meat during the intermediate and late stages of storage, thereby mitigating the accumulation of alkaline substances and inhibiting an increase in pH value [26,27]. The pH value of chilled vacuum-packed lamb packaged with LORP, MORP, and HORP showed a decreasing trend during the later stages of storage, indicating a potential shift towards anaerobic microorganisms as the dominant spoilage bacteria, possessing the ability to degrade carbohydrates and produce organic acids like lactic acid, and so on [28].

### 3.4. Effects of Packaging Barrier Coupled with Storage Temperature on Color in Chilled Lamb During Storage

Color acts as a pivotal indicator of the sensory attributes of uncooked meat and plays a significant role in shaping consumers’ perception and evaluation of meat quality [29]. The effects of packaging barrier coupled with storage temperature on the change in color of chilled lamb is shown in Table 1. With the extension of storage time, there was no statistically significant difference in the L* value between the HORP and LORP groups (*p* > 0.05). During storage, both the a* and b* values of lamb in all groups initially increased and then decreased. The a* value of lamb in the HORP and MORP groups was significantly higher than that in the LORP group, indicating that high- and medium-oxygen-inhibiting packaging materials could effectively inhibit myoglobin oxidation in chilled lamb and control the increase of b* value.

### 3.5. Effects of Packaging Barrier Coupled with Storage Temperature on TVB-N Value in Chilled Lamb During Storage

**Figure 4:**
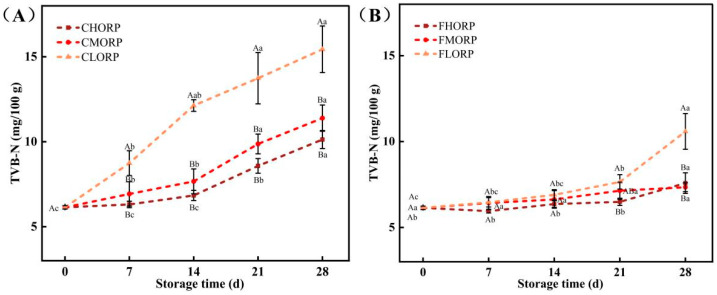
Changes in the TVB-N of chilled lamb under different barrier packaging during storage at 4 °C (**A**) and −1 °C (**B**). Different small letters (a, b, c) indicate a significant difference (*p* < 0.05) in storage time; at the same time point, different capital letters (A, B) indicate a significant difference (*p* < 0.05) among the treatments.

The main components of TVB-N are primarily ammonia and amines, which are produced through the degradation of animal protein and other nitrogen-containing compounds [30]. The initial TVB-N value of the chilled lamb was 6.14 mg/100 g. The TVB-N content in chilled lamb exhibited an increasing trend with the rise in oxygen-permeability rate of the packaging film, while a decrease in storage temperature significantly suppressed the increased rate of TVB-N content, as shown in Figure 4A,B. The CLORP group exhibited the highest rate of increase among the groups, reaching 15.44 mg/100 g at 28 d and surpassing the GB2707-2016 15 mg/100 g. However, the TVB-N content of the CHORP and CMORP groups at 28 d were 10.13 and 11.39 mg/100 g, significantly lower than the CLORP group (15.44 mg/100 g). The TVB-N content in the FHORP, FMORP, and FLORP groups was determined to be 7.34, 7.58, and 10.59 mg/100 g, respectively, at the conclusion of the 28-day storage period, exhibiting a significant decrease compared to that observed at 4 °C. The above results are consistent with the TCV result. The *Pseudomonas* and *Brochothrix* strains have been reported as the predominant bacterium responsible for protein decomposition in meat. Through a comparison of various barrier packaging and storage temperatures, it was determined that *Pseudomonas* and *Brochothrix* are the primary bacteria causing the decrease in freshness and spoilage in meat. The above results once again demonstrate that the utilization of a preservation method involving high-barrier packaging coupled with storage at a temperature of −1 °C represents an effective approach for extending the shelf life of chilled lamb.

### 3.6. Effects of Packaging Barrier Coupled with Storage Temperature on Total Sulfhydryl Content in Chilled Lamb During Storage

**Figure 5:**
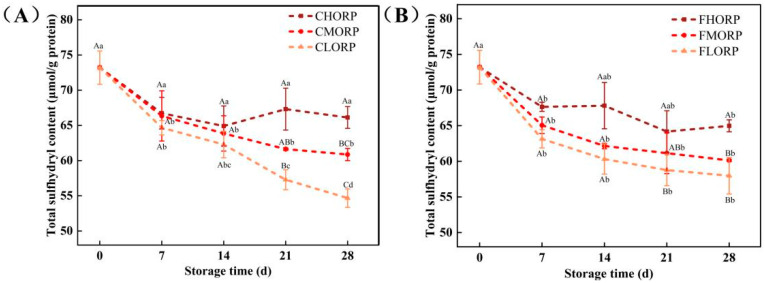
Changes in the total sulfhydryl content of chilled lamb under different barrier packaging during storage at 4 °C (**A**) and −1 °C (**B**). Different small letters (a, b, c) indicate a significant difference (*p* < 0.05) in storage time; at the same time point, different capital letters (A, B, C) indicate a significant difference (*p* < 0.05) among the treatments.

The alteration in sulfhydryl content serves as a crucial indicator for assessing the interaction between compounds and protein oxidation reactions, reflecting the oxidative state of the protein [31]. The variations in the total sulfhydryl content of chilled lamb among different treatment groups are illustrated in Figure 5. During the storage, a decrease in sulfhydryl content was observed in chilled lamb, which exhibited an inverse correlation with TVB-N levels. However, both the changes in sulfhydryl content and TVB-N value confirmed the occurrence of protein oxidation and decomposition during the storage process of chilled lamb [32]. As shown in Figure 5A,B, the changes in the sulfhydryl group content of chilled lamb, packaged with different levels of oxygen permeability during the storage period are as follows: LORP > MORP > HORP. The sulfhydryl content of chilled lamb stored at 4 °C was observed to be lower than that stored at −1 °C during the same period, indicating a temperature-dependent variation. This further suggests that the oxidation process of proteins can be more effectively regulated by the combined influence of oxygen concentration in the packaging and storage temperature, leading to the conversion of -SH groups into disulfide compounds in peptides, ultimately resulting in a deterioration of the quality and sensory attributes of fresh lamb products [33,34].

### 3.7. Effects of Packaging Barrier Coupled with Storage Temperature on Carbonyl Content in Chilled Lamb During Storage

**Figure 6:**
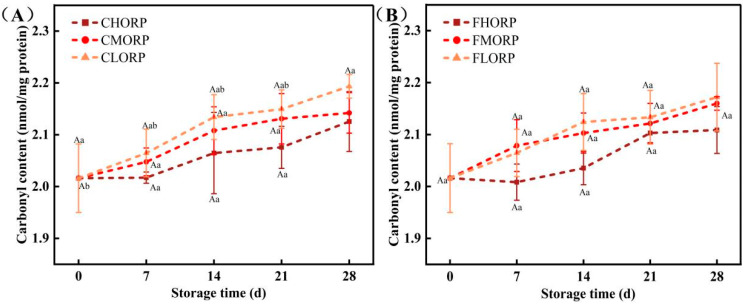
Changes in the carbonyl content of chilled lamb under different barrier packaging during storage at 4 °C (**A**) and −1 °C (**B**). Different small letters (a, b) indicate a significant difference (*p* < 0.05) in storage time; at the same time point, different capital letters (A) indicate a significant difference (*p* < 0.05) among the treatments.

The carbonyl value primarily indicates the extent of protein oxidation [32]. During this process, amino acid residues in protein side chains may undergo oxidative reactions, resulting in the formation of carbonyl compounds, which subsequently bind to proteins, leading to modifications in protein structure and function, ultimately influencing the quality of chilled meat [35]. The changes in the carbonyl content of chilled lamb in different treatment groups are shown in Figure 6. The carbonyl content of total protein exhibited an upward trend in each treatment group as the storage time was prolonged, with no statistically significant differences observed among all groups (*p* > 0.05). The initial carbonyl content of lamb was measured to be 2.02 nmol/mg on day 0. The increase in the packaging barrier can effectively reduce the degree of protein oxidation and inhibit the formation of carbonyl compounds in chilled lamb, as evidenced by comparing the carbonyl value of lamb stored in packages with different oxygen permeability rates during storage. Comparing the impact of storage temperature and packaging barrier on the carbonyl value of chilled lamb, it is evident that both factors have equivalent effects. The results indicate that a storage temperature of −1 °C is more conducive to lamb preservation. After 28 d, the carbonyl contents increased to 2.13 nmol/mg for the CHORP group and 2.11 nmol/mg for the FHORP group, while the CLORP group had the highest level at 2.19 nmol/mg. The carbonyl value of meat has been found to exhibit a strong correlation with its flavor profile [36]. As a crucial parameter for assessing protein oxidation in meat products, any changes in the carbonyl value will directly impact the formation of volatile compounds [36,37].

### 3.8. Effects of Packaging Barrier Coupled with Storage Temperature on Volatile Odor in Chilled Lamb During Storage

**Figure 7:**
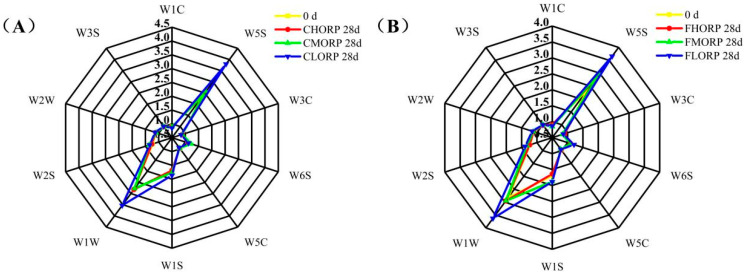
Changes in the volatile odor of chilled lamb under different barrier packaging during storage at 4 °C (**A**) and −1 °C (**B**). W1C: sensitive to aromatic components, benzene; W1W: sensitive to hydrogen sulfide; W3C: sensitive to aromatic components, ammonia; W3S: sensitive to long-chain alkanes; W6S: mainly sensitive to hydrogen; W2W: sensitive to aromatic compounds and organic sulfides; W5C: sensitive to short-chain alkanes and aromatics; W5S: sensitive to nitrogen oxides.

The presence of protein, fatty acids, and other precursors in meat can generate flavor compounds, while excessive oxidation may result in the formation of undesirable flavors such as hexaldehyde, sulfide, putrescine, cademine, and other substances [32,38,39]. The changes in the volatile odor of chilled lamb among different treatment groups were analyzed by electronic noses, and the results are shown in Figure 7. The W1W and W5S sensors, which are capable of sensing sulfides and nitrogen oxides, have the strongest response values when analyzing samples throughout the storage process. The study by Bassey et al. [40] demonstrated the sample’s sensitivity to the W1W sensor, indicating significant instances of corruption. The response values of sensors W1C, W3C, W3S, W6S, W2W, and W5C did not exhibit any significant differences among the three groups. Consistent with the results of the effects of packaging-barrier properties and storage temperature on protein oxidation, high-barrier packaging can effectively reduce the formation of sulfide and nitrogen oxides in chilled lamb during storage.

### 3.9. Effects of Packaging Barrier Coupled with Storage Temperature on Water Phase Change in Chilled Lamb During Storage

**Figure 8:**
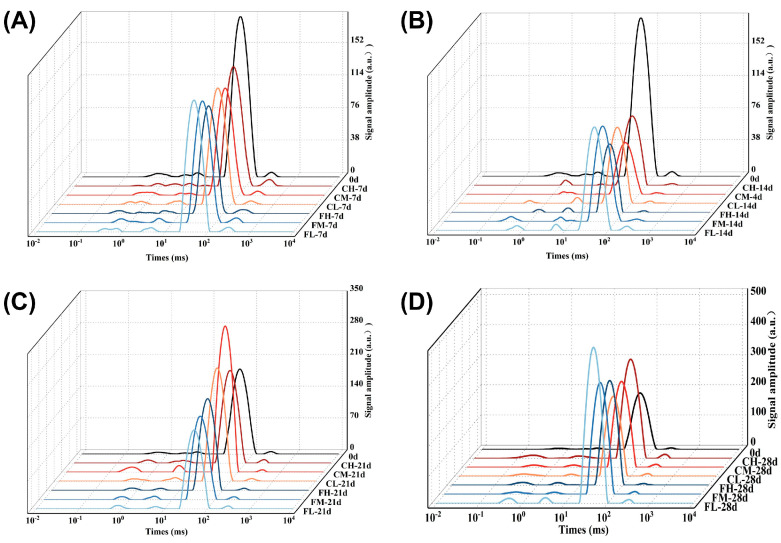
Changes in the water phase of lamb freshness under different barrier packaging during storage at 4 °C and −1 °C. T_2_ relaxation time: 7 d (**A**), 14 d (**B**), 21 d (**C**), and 28 d (**D**).

The presence of water in muscle tissue manifests itself in the forms of free water, bound water, and immobile water [41]. Prolonged storage can give rise to oxidative reactions involving proteins and other substances, resulting in detrimental effects on the myofibrillar structure, heightened permeability of the cell membrane, and subsequent loss of free water, which also induces alterations in the state of immobile water, ultimately exerting a significant impact on the quality of fresh meat [25,41,42]. The LF-NMR technique is highly effective for the nondestructive quantification of water distribution and mobility in muscle and meat [43,44]. Guo et al. [45] revealed that the proton transverse relaxation time T_2_ can effectively assess alterations in water distribution and mobility within meat tissues during curing at various ultrasonic frequencies. The water phase composition of chilled lamb in different treatment groups is shown in Figure 8 during storage and preservation. The relaxation time of both bound water and free water demonstrated a progressive anteposition across all treatment groups with increasing storage time, indicating a gradual attenuation in the interaction between water and protein as well as a reduction in water content. The −1 °C group demonstrated reduced levels of free water content and increased levels of immobile water content in lamb compared to the 4 °C group during the later stage of storage (28 d). The FHORP group encountered less difficulty in converting immobile water into free water and exhibited superior moisture-retention capabilities, highlighting the contribution of HORP material to enhanced moisture preservation in chilled lamb.

## 4. Conclusions

This investigation focused on the optimal barrier packaging and storage temperature for preserving chilled lamb, achieved through a comprehensive combination of three types of oxygen-permeability packaging with two distinct storage temperatures. The results showed that the storage of chilled lamb with oxygen permeability rates of lower than 1.70 cm^3^/(m^2^·24 h·0.1 MPa) and a storage temperature of −1 °C could significantly inhibit the growth and propagation of dominant spoilage bacterias *Pseudomonas* and *Brochothrix* and the increase of TVC. After 28 days of storage, the TVC of the FHORP was only 6.20 log CFU/g, the TVB-N was only 7.34 mg/100 g, and the sulfhydryl content decreased at a lower rate than other groups. The growth rate in the carbonyl value of the FHORP was the lowest. The results of the electronic nose showed that a storage temperature of −1 °C coupled with oxygen permeability lower than 1.70 cm^3^/(m^2^·24 h·0.1 MPa) was more conducive to inhibiting the formation of sulfide and nitrogen oxidation, affecting the flavor perception of chilled lamb meat. In conclusion, packaging with an oxygen transmission rate below 1.70 cm^3^/(m^2^·24 h·0.1 MPa) in conjunction with a storage temperature of −1 °C is a potential and effective preservation method to extend the shelf life of fresh lamb to 28 days, through a combination of reduced oxygen levels and low-temperature conditions to achieve the inhibition of microbial growth and enzyme activity.

## Figures and Tables

**Table 1:** Changes in the color of chilled lamb under different barrier packaging during storage at 4 °C and −1 °C.

Storage Time (d)		4 °C			−1 °C		
		CHORP	CMORP	CLORP	FHORP	FMORP	FLORP
L*	0	40.63 ± 0.61 ^Aa^	40.63 ± 0.61 ^Ab^	40.63 ± 0.61 ^Ab^	40.63 ± 0.61 ^Aab^	40.63 ± 0.61 ^Aa^	40.63 ± 0.61 ^Ab^
	7	42.13 ± 0.86 ^Aa^	41.42 ± 0.69 ^Aab^	42.85 ± 0.87 ^Aa^	41.90 ± 0.69 ^Aa^	40.84 ± 0.78 ^Aa^	40.97 ± 0.74 ^Aab^
	14	41.96 ± 0.45 ^Aa^	40.33 ± 0.44 ^BCb^	41.02 ± 0.39 ^ABCab^	39.73 ± 0.54 ^Cb^	40.17 ± 0.66 ^Ca^	41.79 ± 0.69 ^ABab^
	21	42.50 ± 0.55 ^Aa^	41.91 ± 0.64 ^Aab^	41.87 ± 0.55 ^Aab^	41.30 ± 0.71 ^Aab^	41.22 ± 0.63 ^Aa^	41.15 ± 0.53 ^Aab^
	28	41.51 ± 0.39 ^ABa^	42.90 ± 0.85 ^Aa^	42.47 ± 0.57 ^ABab^	41.13 ± 0.34 ^ABab^	40.93 ± 0.69 ^Ba^	42.65 ± 0.77 ^ABa^
a*	0	18.26 ± 0.51 ^Ab^	18.26 ± 0.51 ^Ab^	18.26 ± 0.51 ^Aa^	18.26 ± 0.51 ^Ab^	18.26 ± 0.51 ^Ab^	18.26 ± 0.51 ^Aa^
	7	19.70 ± 0.47 ^Aab^	19.84 ± 0.43 ^Aa^	14.78 ± 0.90 ^Cbc^	18.86 ± 0.45 ^Ab^	19.66 ± 0.36 ^Aa^	17.08 ± 0.91 ^Ba^
	14	20.09 ± 0.62 ^ABa^	18.66 ± 0.26 ^Bb^	16.63 ± 0.58 ^Cab^	20.68 ± 0.36 ^Aa^	18.90 ± 0.41 ^Bab^	12.34 ± 0.74 ^Db^
	21	19.17 ± 0.30 ^Bab^	18.44 ± 0.32 ^Bb^	15.93 ± 0.47 ^Cbc^	21.05 ± 0.42 ^Aa^	18.77 ± 0.57 ^Bab^	12.45 ± 1.19 ^Db^
	28	17.90 ± 0.84 ^Ab^	17.42 ± 0.41 ^Ab^	14.53 ± 0.59 ^Bc^	18.36 ± 0.44 ^Ab^	17.76 ± 0.49 ^Ab^	13.06 ± 1.07 ^Bb^
b*	0	9.54 ± 0.40 ^Ac^	9.54 ± 0.40 ^Ac^	9.54 ± 0.40 ^Ac^	9.54 ± 0.40 ^Ac^	9.54 ± 0.40 ^Ac^	9.54 ± 0.40 ^Ac^
	7	11.16 ± 0.31 ^ABab^	11.41 ± 0.32 ^ABa^	11.16 ± 0.39 ^Aa^	10.44 ± 0.36 ^Bc^	10.83 ± 0.31 ^ABab^	11.65 ± 0.52 ^Ab^
	14	11.76 ± 0.56 ^Ba^	10.90 ± 0.28 ^BCab^	11.36 ± 0.41 ^Ba^	11.72 ± 0.23 ^Bb^	10.21 ± 0.24 ^Cbc^	13.93 ± 0.36 ^Aa^
	21	11.04 ± 0.34 ^BCDab^	10.46 ± 0.33 ^CDabc^	9.94 ± 0.41 ^Dbc^	12.78 ± 0.54 ^Aa^	11.49 ± 0.66 ^BCa^	11.96 ± 0.55^ABb^
	28	10.30 ± 0.24 ^Bbc^	9.99 ± 0.37 ^Bbc^	10.82 ± 0.48 ^Bab^	9.93 ± 0.27 ^Bc^	10.30 ± 0.24 ^Bbc^	11.94 ± 0.46 ^Ab^

Values represent means ± SE (*n* = 6). For the same treatments, different small letters (a, b, c) indicate a significant difference (*p* < 0.05) in storage time; at the same time point, different capital letters (A, B, C, D) indicate a significant difference (*p* < 0.05) among the treatments. Abbreviations: CHORP: stored at 4 °C and treated with high-oxygen-resistant packaging; CMORP: stored at 4 °C and treated with medium-oxygen-resistant packaging; CLORP: stored at 4 °C and treated with oxygen-resistant packaging; FHORP: stored at −1 °C and treated with high-oxygen-resistant packaging; FMORP: stored at −1 °C and treated with medium-oxygen-resistant packaging; and FLORP: stored at −1 °C and treated with oxygen-resistant packaging.

## Data Availability

The data presented in this study are available on request from the corresponding author. The data are not publicly available as they are incorporated into other ongoing research and should be protected until formal publication.
